# Anti-Obesity Natural Products Tested in Juvenile Zebrafish Obesogenic Tests and Mouse 3T3-L1 Adipogenesis Assays

**DOI:** 10.3390/molecules25245840

**Published:** 2020-12-10

**Authors:** Hiroko Nakayama, Kanae Hata, Izumi Matsuoka, Liqing Zang, Youngil Kim, Djongchi Chu, Lekh Raj Juneja, Norihiro Nishimura, Yasuhito Shimada

**Affiliations:** 1 Graduate School of Regional Innovation Studies, Mie University, Tsu 514-8507, Japan; 27293301@m.mie-u.ac.jp (H.N.); kanae.hata.kh@gmail.com (K.H.); 27220620@m.mie-u.ac.jp (I.M.); liqing@doc.medic.mie-u.ac.jp (L.Z.); nishimura.norihiro@mie-u.ac.jp (N.N.); 2 Zebrafish Drug Screening Center, Mie University, Tsu 514-8507, Japan; 3 Rohto Pharmaceutical Co., Ltd, Osaka 544-0012, Japan; youngil@rohto.co.jp (Y.K.); ssj3590@yahoo.co.jp (D.C.); juneja@rohto.co.jp (L.R.J.); 4 Department of Integrative Pharmacology, Mie University Graduate School of Medicine, Tsu 514-8507, Japan; 5 Department of Bioinformatics, Mie University Advanced Science Research Promotion Center, Tsu 514-8507, Japan

**Keywords:** drug discovery, diabetes, metabolic syndrome, screening

## Abstract

(1) Background: The obesity epidemic has been drastically progressing in both children and adults worldwide. Pharmacotherapy is considered necessary for its treatment. However, many anti-obesity drugs have been withdrawn from the market due to their adverse effects. Instead, natural products (NPs) have been studied as a source for drug discovery for obesity, with the goal of limiting the adverse effects. Zebrafish are ideal model animals for in vivo testing of anti-obesity NPs, and disease models of several types of obesity have been developed. However, the evidence for zebrafish as an anti-obesity drug screening model are still limited. (2) Methods: We performed anti-adipogenic testing using the juvenile zebrafish obesogenic test (ZOT) and mouse 3T3-L1 preadipocytes using the focused NP library containing 38 NPs and compared their results. (3) Results: Seven and eleven NPs reduced lipid accumulation in zebrafish visceral fat tissues and mouse adipocytes, respectively. Of these, five NPs suppressed lipid accumulation in both zebrafish and 3T3-L1 adipocytes. We confirmed that these five NPs (globin-digested peptides, green tea extract, red pepper extract, nobiletin, and Moringa leaf powder) exerted anti-obesity effects in diet-induced obese adult zebrafish. (4) Conclusions: ZOT using juvenile fish can be a high-throughput alternative to ZOT using adult zebrafish and can be applied for in vivo screening to discover novel therapeutics for visceral obesity and potentially also other disorders.

## 1. Introduction

Obesity, especially abdominal obesity, which is characterized by increased visceral adipose tissue (VAT), has been distinctly linked to metabolic diseases including hepatosteatosis, type 2 diabetes mellitus (T2DM), atherosclerosis, cardiovascular diseases, and several types of cancers. Dietary therapies (hypocaloric diet with low fat, low carbohydrate, and/or high-fibers) and exercise are the first-line treatment to reduce VAT; however, their success rates are lower than 25% [1]. Instead, anti-obesity medications such as weight loss and anorexigenic drugs are used for patients with severe obesity including those suffering from hypertension and T2DM [2]. Although pharmacological management is more effective than diet or exercise therapy, many drugs are not approved or have been withdrawn from the market because of their adverse effects such as the CB1 receptor agonist rimonabant, which imposed a heightened risk of psychiatric diseases [3,4]. For this reason, a variety of natural products (NPs) and their constituents are being tested to ameliorate visceral obesity with minimal side effects [5].

NPs include thousands of molecules synthesized by living organisms (e.g., plants, bacteria, fungi) that resemble secondary metabolites. To discover anti-obesity NPs and their active constituents, cell-based screening such as adipogenesis assays using mouse 3T3-L1 preadipocytes are generally used. Several constituents of NPs such as polyphenols and other phytochemicals were shown to reduce lipid markers of obesity and to reduce the expression of lipid-modifying gene products such as proliferator-activated receptor-gamma using 3T3-L1 cells [6,7]. After finding promising compounds in vitro, conventional in vivo studies are performed in rodents to predict their clinical efficacies. Testing in rodents requires a relatively large amount of compounds, especially for the treatment of chronic diseases such as obesity. Thus, it is necessary to prepare a large volume of bioactive constituents by the fractionation of drug-like NPs, which is a bottleneck in NP-based drug discovery. In addition, because obesity is a complex and systemic disease, in vitro results are not always translatable to clinical situations with respect to drug efficacy, drug delivery, absorption, pharmacokinetics, and side effects.

To reduce the gap between in vitro studies and preclinical assessments in animals, zebrafish are becoming an important model animal for drug testing. Zebrafish, a small vertebrate fish, are used in the drug discovery process because of their high productivity, ease of animal welfare management, suitability for in vivo imaging, the similarity of their genome structure to that of the humans, and the efficiency of genome editing [8]. Zebrafish VAT is histologically [9] and transcriptionally [10,11] similar to that of humans. We previously developed a diet-induced obesity (DIO) model of zebrafish for the first time [10] and discovered several natural products with lipid-lowering and/or visceral adipose-reducing properties [5,12,13]. DIO-zebrafish are a suitable model to evaluate drug response and analyze obese pathologies for human obesity; however, around 100 fish can be produced per month, which is not suitable for screening studies [14]. Tingaud-Sequeira et al. first reported that young zebrafish (up to four weeks post fertilization) exposed to boiled chicken eggs showed an increase in visceral adiposity detected by live imaging, named the zebrafish obesogenic test (ZOT) [15]. Their increased VATs were reduced by short-time exposure to phenylephrine, an alpha-adrenergic agonist [15], and green tea extracts [16]. Due to the small size of the young zebrafish, ZOT can be performed in a 6-well plate format; however, there is no systematic analysis to compare the results between ZOT and cell-based assays. Here, we performed the ZOT and 3T3-L1 adipogenesis assay for 38 promising NPs expected to reduce obesity and compared their results. In addition, we tested NPs in adult DIO-zebrafish to validate the results.

## 2. Results

### 2.1. Zebrafish Obesogenic Test (ZOT) Identifies Seven Anti-Obese Natural Products (NPs)

**Figure 1:**
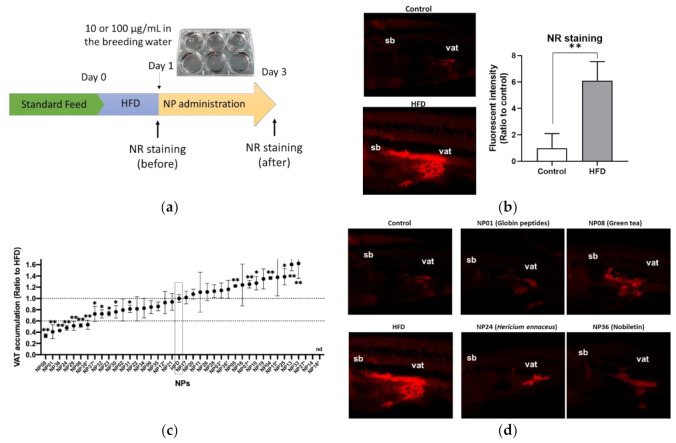
Zebrafish obesogenic test (ZOT) identifies seven anti-obese natural products (NPs). (**a**) Experimental design for ZOT. (**b**) Short term high-fat diet (HFD) increased the amount of visceral adipose tissue (VAT). Right panel indicates typical images of the control and HFD groups. Red indicates Nile Red (NR)-stained VAT. sb; swimming bladder, vat; visceral adipose tissue. Left panel indicates quantification of NR stained area. Values presented are means. Error bars indicate SD. *n* = 5, ** *p* < 0.01, Student’s t-test. (**c**) ZOT result. Dotted lines indicate 1 and 0.6 ratio to HFD alone group (HFD). NPs with asterisks were administered at lower volumes (10 µg/mL) because of their toxicity. The others were administered at 100 µg/mL in the breeding water. Values presented are means. Error bars indicate SD. *n* = 5–10, * *p* < 0.05, ** *p* < 0.01 vs. control, one-way ANOVA with Bonferroni–Dunn multiple comparison. nd indicates not determined because of high toxicity (NP18). (**d**) Typical image of effective NPs in (**c**).

We first performed ZOT to identify anti-obese NPs using juvenile zebrafish (four to six weeks post fertilization, about 1 cm body length). The tested library contained 38 NPs, which were expected to improve obesity based on previous studies (Table S1). The experimental design is shown in Figure 1a. After a 1-day high-fat diet feeding (HFD), Nile Red (NR) staining was performed (day 1). The NR-stained visceral adipose tissue (VAT; red color) was significantly (*p* < 0.01) increased in zebrafish fed a HFD compared to those fed a normal diet (ND; Figure 1b), as shown previously [16]. Then, the zebrafish were exposed to test the NPs (100 μg/mL) for 48 h, and NR imaging was conducted again (day 3) to compare the NR intensities of the VAT before and after treatment (day 3/day 1). Nine NPs showed toxicity (dead or fish not swimming), so we administered them at a lower concentration of 10 μg/mL. Only NP18 (allyl isothiocyanate from Japanese mustard) was toxic even at a lower concentration (1 μg/mL), so we did not pursue it further. As a result, seven of 38 tested NPs (NP01; globin digested peptides (GDP), NP06; beetroot extract, NP08; green tea extract (GTE), NP24; *Hericium erinaceus* (HE) powder, NP29; red pepper extract, NP36; nobiletin and NP38; *Moringa olefera* powder) significantly (*p* < 0.01) reduced the VAT by over 60% compared to HFD alone (Figure 1c). The typical images of effective NPs (NP01, NP08, NP24, and NP38) are shown in Figure 1d.

### 2.2. Mouse Adipocyte Screenings Identifies 11 Lipid-Lowering Compounds

**Figure 2:**
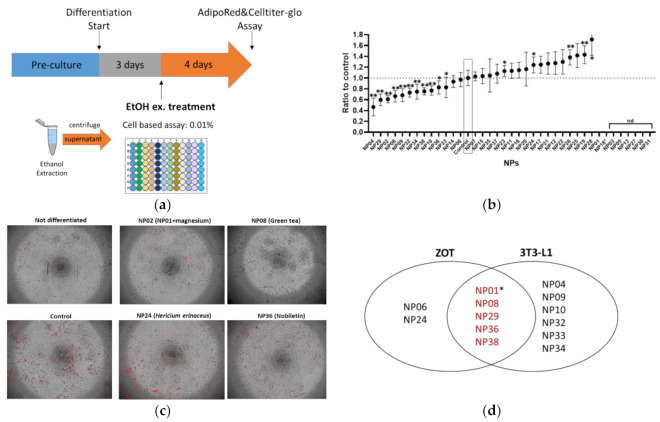
Mouse 3T3-L1 preadipocyte differentiation assay identified ten lipid-lowering NPs. (**a**) Experimental design for 3T3-L1 assay. (**b**) Result of 3T3-L1 screening. NPs without data (not determined: ND) were due to inability to extract compounds with ethanol extraction. Values presented are means. Error bars indicate SD. *n* = 8, * *p* < 0.05, ** *p* < 0.01 vs. control l, one-way ANOVA with Bonferroni–Dunn multiple comparison. (**c**) Representative images of differentiated 3T3-L1 cells with the same NPs as in Figure 1d. Red indicates lipid accumulation. (**d**) Venn diagram analysis of the effective NPs in both the ZOT (Figure 1) and 3T3-L1 cell-based assay. * While NP01 (globin digested peptides [GDP]) could not be evaluated in the 3T3-L1 assay, NP02 (GDP with magnesium) reduced lipid accumulation in 3T3-L1 cells. Thus, we categorized NP01 as effective in both assays.

Mouse 3T3-L1 adipocytes are a widely used in vitro model of white adipocyte differentiation. To compare the results between zebrafish and mouse adipocytes, we performed an adipogenesis assay using the 3T3-L1 cells for the same NP library used for the ZOT. As shown in Figure 2a, three days after adipocyte differentiation started, ethanol extracts of NPs were administered to the cells for four days, followed by AdipoRed staining. As a result, 11 NPs significantly (*p* < 0.05) suppressed lipid accumulation in 3T3-L1 cells (Figure 2b). There was no significant effect on cell viability after treatment with the NPs, except NP18 (allyl isothiocyanate) was the same as the ZOT result. As some NPs could not be extracted by an equal volume of ethanol, their results are presented as “not determined (nd)”. Representative images of the differentiated adipocytes with or without the NPs (NP02 (GDP with magnesium), NP08 (GTE), NP24 (HE, effective only in ZOT not in 3T3-L1 cells), and NP38 (nobiletin)) are depicted in Figure 2c. Venn diagram analysis revealed that four NPs (NP08, NP29, NP36, and NP38) were able to suppress lipid accumulation in both zebrafish larvae and mouse adipocytes (Figure 2d). In addition, while NP01 (GDP alone) could not be evaluated in 3T3-L1 cells because it was poorly extracted by ethanol, NP02 and GDP with minerals (mainly magnesium) significantly (*p* < 0.05) suppressed lipid accumulation. Thus, we concluded that five NPs had common effects in the ZOT and 3T3-L1 assays.

### 2.3. Validation Study Using Diet-Induced Adult Zebrafish

**Figure 3:**
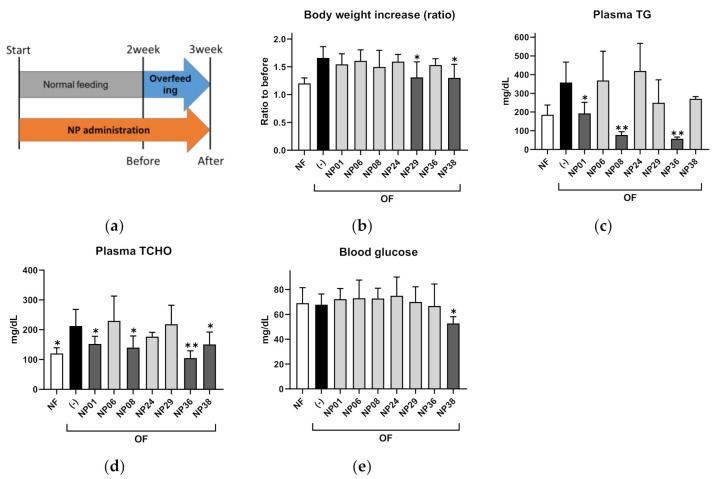
NP administration in adult diet-induced obese (DIO) zebrafish. (**a**) Experimental design for DIO-zebrafish experiment. (**b**) Body weight increases in DIO-zebrafish with or without NPs. (**c**–**e**) Plasma triglycerides (**c**), total cholesterol (**d**), and fasting blood glucose (**e**) at the end of the experiment. Values presented are means. Error bars indicate SD. *n* = 5–10, * *p* < 0.05, ** *p* < 0.01 vs. control, one-way analysis of variance (ANOVA) with Bonferroni–Dunn multiple comparison.

To validate the results of the ZOT, we performed a feeding experiment for the seven hit NPs using adult DIO-zebrafish (Figure 3a). After 1-week of overfeeding (OF), red pepper extract (NP29), and Moringa (Moringa oleifera) leaf powder (NP38) significantly (*p* < 0.05) suppressed body weight increases compared to the control group (Figure 3b). NP01 (GDP), NP08 (GTE), and NP36 (nobiletin) reduced the increase in plasma triglycerides (TG; Figure 3c; *p* < 0.05 or *p* < 0.01 vs. OF group) in DIO-zebrafish. Plasma total cholesterol (TCHO) showed a similar tendency to plasma TG (Figure 3d). Only NP38 decreased fasting blood glucose compared to the OF group (Figure 3e; *p* < 0.01). NP06 (beetroot extract) and NP24 (HE) did not show any effect in DIO-zebrafish. The results of the ZOT, 3T3-L1 assay, and DIO-zebrafish are summarized in Table 1.

## 3. Discussion

### 3.1. Anti-Obese NPs Identified in this Study

In this study, we compared two types of screening protocols, the mouse 3T3-L1 cell-based in vitro differentiation assay and the zebrafish obesogenic test (ZOT), using the same NP library. As shown in Figure 2, five NPs (NP01, NP08, NP29, NP36, and NP38) exerted common anti-obesity effects in the ZOT and 3T3-L1 cell-based assays. The five NPs also exhibited anti-obesity effects in an adult zebrafish model of obesity (Table 1).

NP01 (GDP), which contains several short peptides from porcine globin, has lipid-lowering and anti-diabetic effects in mouse models [17,18]. Our results indicate that GDP has lipid-lowering effects in 3T3-L1 cells and zebrafish models, similar to mouse and human studies. As our zebrafish model did not have increased fasting blood glucose levels during the feeding experiment, we could not evaluate the anti-diabetic effect of GDP and other NPs in this study. Further studies using diabetic zebrafish models [19,20] could evaluate the effects of GDP on blood glucose dysregulation.

NP08 (GTE) is well-known for its anti-obesity effects through its bioactive constituents such as (-)-epigallocatechin-3-gallate and (-)-epigallocatechin in rodent models [21,22,23]. Additionally, several studies have demonstrated the anti-obesity effects of GTE in zebrafish models [13,16,24], which are consistent with our results. In humans, continuous ingestion of a GTE significantly reduces body weight and body fat mass [25], while Huang et al. reported that GTE reduced only serum low-density lipoprotein cholesterol, and did not affect body weight or other blood lipids [26]. These results indicate that there is a difference between rodents, humans, and probably zebrafish. Further studies are needed to elucidate this difference.

NP29, a red pepper extract, is also known for its anti-obesity effects through its bioactive constituent, capsaicin. Capsaicin is a transient receptor potential vanilloid type-1 agonist that suppresses adipogenesis in 3T3-L1 cells and mouse models [27,28], and red pepper (chili) consumption can improve the risk factors for human obesity [29,30]. In our study, NP29 showed lipid-lowering effects in plasma TG and TCHO. Interestingly, in contrast to the previous two NPs (NP01 and NP08), NP29 also suppressed body weight increases in DIO-zebrafish, indicating that NP29 could enhance systemic energy expenditure as well as VAT.

NP36, a citrus peel flavonoid nobiletin, is a promising natural compound used against various human diseases including obesity, has lipid-lowering effects, and can improve insulin resistance [31,32,33,34]. We also detected the lipid-lowering effects of NP36 in adult zebrafish (Table 1), while its anti-diabetic properties could not be evaluated, similar to NP01 (GDP). Choi et al. reported that nobiletin downregulates gene expression related to adipogenesis including peroxisome proliferator-activated receptor γ (*Pparg*) and CCAAT/enhancer binding protein α (*Cebpa*) in mouse 3T3-L1 cells [34], which are also involved in adipogenesis in zebrafish [35]. Thus, the anti-obesity mechanism of NP36 could be common between mammals and zebrafish.

The last effective NP, NP38, is *Moringa oleifera* leaf powder, which is also well-known for its multi-medicinal use including its use in obesity and related diseases [36,37]. As an anti-obesity NP, NP38 has been reported to have a variety of effects such as a reduction in weight gain, improvement in insulin resistance, and suppression of glucogenesis and lipid accumulation [38,39,40]. Corresponding with these reports, NP38 improved all obese phenotypes in this study including lowering blood glucose (BG) levels in adult fish. As insulin resistance was not established in this study, the BG-lowering effect of NP38 could be caused by the inhibition of glucogenesis in the liver, as reported in a mouse study [39].

### 3.2. Possible Reasons for the Difference in NP Responses in the Experimental Models

Of the seven NPs selected from the ZOT, NP06 and NP24 did not show any positive effects in DIO-zebrafish and 3T3-L1 cells. NP06, a beetroot extract, has been reported to ameliorate hyperlipidemia in rat models [41] and hypertension in elderly and overweight populations [42]. However, beetroot is thought to function as a nitrate supplier and to reduce inflammation and improve cardiovascular, liver, and metabolic function in rats with metabolic syndrome [43], suggesting that its lipid-lowering effects in mammals are not prominent, similar to our in vivo results. NP24, *Hericium erinaceus*, is a mushroom also called “Lion mane”. It is an edible medicinal mushroom used to treat mild cognitive impairment [44] and cancers [45,46]. NP24 suppresses body weight increases and serum and hepatic TG levels, although only two studies have reported these results [47,48]. One problem with NP efficacies is that the ingredients can change depending on the culture conditions (temperature, nutrient environment, etc.) and the extraction methods. In this study, we chose ethanol extraction for the cell-based experiments and whole NP administration in the in vivo studies, which could be modified through the zebrafish digestive system. This may explain why NP24 was not effective in the DIO-zebrafish study. On the other hand, 54.5% (6 of 11) of 3T3-L1 hit-NPs were not detected in ZOT (Figure 2d). Due to several limitations in cell-based assays such as absorption, distribution, metabolism, and excretion [49], the 3T3-L1 cell-based assay could result in false positives.

From our limited results, there are three possible reasons for the differences between the 3T3-L1 cell-based assay, ZOT, and DIO-zebrafish studies: (1) Due to the relatively short duration of cell-based assays and ZOT, some of the results obtained were not consistent with long-term studies in adult animals; (2) pharmacokinetics and liver metabolism are more advanced in adult zebrafish (DIO-zebrafish) than in 3T3-L1 cells and juvenile zebrafish, so the NPs may not have sufficient efficacy in DIO-zebrafish study (hence, the 3T3-L1 cell-based assay is more sensitive than ZOT); (3) similar to (2), the zebrafish gut flora may be involved in the metabolic absorption of the administrated NPs. The gut flora of zebrafish is different between young and adult zebrafish [50], which would result in reduced absorption of various nutrients and components. In addition, there is a limitation in ZOT and DIO-zebrafish compared to the mouse model. As fish are poikilotherms, NPs that induce thermogenesis subsequent to activation of brown (or beige) adipose tissues cannot be evaluated in zebrafish-based methodologies. Of course, to step up the clinical trial, we have to perform mouse studies to determine the equivalent human dosage.

## 4. Materials and Methods

### 4.1. Preparation of the NP Library

Natural products (NPs) were prepared by Rohto Co. Ltd. (Osaka, Japan). The NPs are listed in Table S1. For cell-based experiments, the extracts were prepared as follows: (1) Test samples (100 mg) were mixed with 100 µL ethanol (EtOH) and vortexed; (2) Samples were left to rest for 10 min and then centrifuged (13,000× *g*, 5 min) to collect the supernatant (stored at −80 °C); (3) One microliter of supernatant was transferred to a 96-well plate and EtOH was removed by freeze-drying (stored at −80 °C); (4) 100 µL of adipocyte maintenance medium was added just before 3T3-L1 cell administration; and (5) Plates were shaken on a plate shaker for 15–20 min to obtain a cell administration solution.

### 4.2. Zebrafish Obesogenic Test (ZOT)

ZOT was performed as previously described [15] with some modifications [16]. Zebrafish (4–5 week-post-fertilization, standard length ca. 7–9 mm) were assigned to either a high-fat diet (HFD) or a control group with five fish per 500 mL tank. For the HFD group, we prepared 1 g of boiled chicken yolk suspended in 15 mL water as a stock and supplied a 2 mL suspension to each tank twice a day (morning and evening) for one day (day 0). The next day (day 1), zebrafish were stained with 5 μg/mL Nile Red (NR) in 1% acetone–H_2_O for 30 min and washed three times with breeding water (0.3 × Danieau’s solution [17.4 mM NaCl, 0.21 mM KCl, 0.18 mM Ca(NO_3_)_2_, 0.12 mM MgSO_4_, and 1.5 mM HEPES buffer, pH 7.6]) for 10 min. The NR signal was captured using a BZ-X710 fluorescence microscope (TRITC filter; Keyence, Tokyo, Japan) while the fish were under anesthesia (500 ppm, 2-phenoxyethanol; Wako Pure Chemicals, Osaka, Japan). Then, the fish were treated with NPs (100 μg/mL in 0.3 × Danieau’s solution) for 48 h in each tank and stained again with NR to visualize visceral adipose tissue (VAT; day 3). The NR intensity of the VAT was quantified using ZF-Mapper software [51], and the ratio for day 3/day 1 was calculated as previously reported [16]. There was no effect of NR staining on fish survival during ZOT. The concentration of NPs that exhibited toxicity during the experiment was reduced to 10 μg/mL.

### 4.3. T3-L1 Adipocyte Differentiation Assay

Mouse 3T3-L1 preadipocytes were purchased from DS Pharma Biomedical (Osaka, Japan). Preadipocytes were cultured in Dulbecco’s modified Eagle medium—high glucose medium (Gibco, Gaithersburg, MD, USA) supplemented with 10% calf bovine serum (Gibco) and penicillin-streptomycin (Nacalai Tesque, Kyoto, Japan) at 37 °C in a humidified 5% CO_2_ atmosphere until confluent in a 96-well plate format. Two days after confluence (day 0), the cells were stimulated to differentiate into adipocyte differentiation medium (ADM; DS Pharma Biomedical) for three days (day 3). ADM contains 3-isobutyl-1-methylxanthine, dexamethasone, and insulin to stimulate differentiation. Cells were maintained in adipocyte maintenance medium (DS Pharma Biomedical) for an additional four days (day 7), with or without 10 μg/mL NP ethanol extracts. On day 7, intracellular lipid droplets were stained using AdipoRed Assay Reagent (Lonza, Walkersville, MD, USA) according to the manufacturer’s instructions. AdipoRed contains the hydrophilic stain Nile Red. When partitioned in a hydrophobic environment, Nile Red becomes fluorescent. After image capture using a BZ-X710 fluorescence microscope (Keyence, Tokyo, Japan), intracellular lipid accumulation was quantified by the measurement of fluorescence (Ex 485 nm/Em 590 nm) using a Victor2 multilabel plate reader (PerkinElmer, Boston, MA, USA). After the AdipoRed Assay, a cell viability assay was performed using the CellTiter-Glo Luminescent Cell Viability Assay (Promega, Madison, WI, USA) according to the manufacturer’s instructions.

### 4.4. Adult Diet-Induced Obese (DIO)-Zebrafish Experiment

For oral administration of NPs to adult zebrafish, 10% NP-containing zebrafish food was prepared as previously described [52]. Briefly, we mixed NPs into gluten dough, freeze-dried the mixture, and ground the mixture to 700-μm granules. As the gluten granules were flavored with 10% commercial fish food (Hikari Crest Cat; Kyorin, Hyogo, Japan), all NP-containing granules were consumed by zebrafish within 10 min of provision. Female adult zebrafish (3-month-old) were randomly assigned into three treatment groups with five fish per 2 L tank (*n* = 5 per group): the normal feeding group (NF), where zebrafish were fed a normal diet throughout the experimental period (three weeks); and the over-feeding group (OF), where zebrafish were fed a normal diet for the first two weeks and then continuously fed with *Artemia* to induce obesity [10] with or without the tested NP-containing food (250 µg/g BW/day) for one week. NP-containing food was fed to zebrafish 30 min before *Artemia* feeding. During feeding, the tank water flow was stopped for 2 h. Leftover food was removed once daily by vacuuming to avoid water pollution. Body length and body weight of the fish were measured every week. The plasma triglyceride, plasma total cholesterol, and blood glucose levels were measured at the end of the experiment, according to our previous studies [53,54].

### 4.5. Statistics

All results are presented as means with standard deviations. Data were analyzed using the Student’s t-test or analysis of variance with the Bonferroni–Dunn multiple comparison procedure, depending on the number of comparisons. Statistical analyses were performed using GraphPad Prism version 8.4.3 (GraphPad Software, San Diego, CA, USA).

## 5. Conclusions

We performed small-scale screening using the ZOT and 3T3-L1 cell-based assays for NPs with anti-obesity effects and compared their results. As a result, 13% (five of 38) of the tested compounds were found to be effective in reducing VAT accumulation in both models. The advantages and limitations of our three methods (3T3-L1 cells, ZOT, and DIO-zebrafish; Table 2) are summarized below.

## Figures and Tables

**Table 1:** Diet-induced obese (DIO)-zebrafish results for natural products (NPs) from the zebrafish obesogenic test (ZOT).

ID	Name	Body Weight	Plasma TG	Plasma TCHO	FBG
NP01	Globin digested peptides	→	↓	↓	→
NP06	Beetroot extract	→	→	→	→
NP08	Green tea extract	→	↓↓	↓	→
NP24	*Hericium ennaceus* powder	→	→	→	→
NP29	Red pepper extract	↓	→	→	→
NP36	Nobiletin	→	↓↓	↓↓	→
NP38	Moringa leaf powder	↓	→	↓	↓

Yellow IDs indicate NPs positive in both ZOT and 3T3-L1 cell-based assays, and IDs without color indicate NPs positive only in ZOT. Single and double down allows indicate *p* < 0.05 and *p* < 0.01, respectively.

**Table 2:** Advantages and disadvantages of 3T3-L1 cell-based assay, ZOT, DIO-zebrafish, and mouse experiments.

	ZOT	3T3-L1	DIO-Zebrafish	Mouse
**Output**	VAT Toxicity	Intracellular lipid	Body weight, feeding volume, fat bioavailability, VAT (white adipose tissue), blood chemistry, histology	Body weight, feeding volume, fat bioavailability, VAT, brown / beige adipose tissue, blood chemistry, histology
**Duration**	1 week	2 weeks	2–4 weeks	2–6 months
**Throughput (animals/experiment)**	High (100~)	Very high (100~)	Medium (~100)	Low
**Cost**	Low	Very low	Medium	High

## Supplementary Materials

Table S1: NPs used in this study.
